# Supplementary material for: Collaborative intelligence in AI: Evaluating the performance of a council of AIs on the USMLE
Source: PLOS Digit Health. 2025 Oct 9;4(10):e0000787. doi: 10.1371/journal.pdig.0000787 (PMC12510544; doi:10.1371/journal.pdig.0000787)
Supplement: S2 File — (ZIP) [file pdig.0000787.s002.zip › S2 Data Raw data files containing unprocessed question inputs and LLM outputs including transcripts of deliberations/STEP3_Question-24-1_Type-MC-NJ.docx]

**Facilitator** **[Round 1]:** An 88-year-old man with osteoarthritis is brought to the office by his daughter because of a 2-day history of severe low back pain and inability to walk. He says he has not fallen and does not recall any trauma to his back. Five years ago, he underwent bilateral hip replacement. His pain and mobility improved following the operation but have worsened during the past year. He now is most comfortable using a wheelchair for ambulation but is able to ambulate for short distances with a walker and assistance. One year ago, carcinoma of the prostate was diagnosed, but he declined therapy and said, ""I'm an old man. When my time comes, I want to die. Just keep me as comfortable as you can."" Vital signs today are temperature 36.6°C (97.9°F), pulse 88/min, respirations 16/min, and blood pressure 188/66 mm Hg. The patient is grimacing with pain and holding his lower back. Palpation discloses point tenderness over L3 through L4, and S1. X-rays of the lumbosacral spine show lytic lesions and compression fracture. The patient refuses additional diagnostic studies. The daughter fears that her father will be ""abandoned"" if he is not evaluated for additional treatment. Which of the following is the most appropriate next step in response to the daughter's concern? (A) Explain the daughter's concerns to the patient and persuasively present the advantages of further evaluation (B) Explain to the daughter that no significant treatment will result from further testing and recommend having a home health nurse (C) Obtain consultation with an oncologist to assess the patient's need for further testing (D) Reassure the daughter and recommend hospice care consultation (E) Reassure the daughter and recommend transfer of the patient to an assisted living facility

**AI_C:** (1) RESTATING THE QUESTION: The clinical case presents an elderly man with a history of osteoarthritis, bilateral hip replacement, prostate cancer, and newly diagnosed lytic lesions and compression fracture on his spine. He refuses any further diagnostic investigation. His daughter is worried and wants to ensure he receives the necessary care. She seeks advice on what the next steps should be. The potential responses include discussing her concerns with her father, informing her that further testing may not yield substantial treatments, consulting an oncologist, recommending hospice care, or recommending a transfer to an assisted living facility. (2) PLANNING: The primary assumption is that the patient's right to decline further investigations is being respected and the focus is on palliative care. An alternate assumption could be that the daughter's concern might warrant a reassessment of the decision to avoid further treatment and there are advantages to additional diagnostic tests. (3) CLINICAL REASONING: The patient has made it clear that he does not want any further diagnostic tests, which would include therapy for his diagnosed prostate cancer. This could be due to the stress, invasive nature, and potential side-effects of such treatments. His pain is likely caused by compression fractures and lytic lesions, which are common in patients with long-standing cancer, and it seems to be quite severe. It would seem that making him comfortable and addressing his quality of life should be the primary approach. (4) CONCLUDING: Taking the clinical presentation, patient's wishes, and daughter's concerns into consideration, the most appropriate next step would be focusing on palliative care. (5) RESPONSE: Therefore, the answer should be (D) Reassure the daughter and recommend hospice care consultation. Hospice care is designed to provide comfort and support to patients and their families when a life-limiting illness no longer responds to cure-oriented treatments. The focus is on controlling pain and symptoms while keeping the patient as comfortable as possible.

**AI_D:** RESTATING THE QUESTION: The case presents an 88-year-old male diagnosed with prostate cancer a year ago, who declined therapy. Recently, he has been experiencing severe low back pain and an inability to walk, with point tenderness at L3-L4 and S1 vertebrae, as well as lytic lesions and compression fracture seen on lumbosacral spine x-ray. His daughter worries about his father being "abandoned" without further medical evaluation and management. The query is about the most appropriate course of action in response to the patient's daughter's concern. PLANNING: Several assumptions can be made here. The first is that the father's worsening condition might be due to the progression of the untreated prostate cancer. Alternatively, the patient's condition could be solely attributed to comorbidities associated with aging, such as osteoarthritis, osteoporosis, deterioration from previous surgeries (hip replacements), or indeed a combination of all. However, based on the clinical progression, the possibility of metastasis of the untreated prostate cancer to his spine causing these symptoms is a high clinical priority. CLINICAL REASONING: Prostate cancer is known for its propensity to metastasize to the bone, and the spine is a common site. Put together with the patient's declining mobility over the past year, the back pain, his refusal for therapy, and the recent imaging findings, these circumstances strongly suggest bony metastasis of the prostate cancer. This presentation is further supported by the patient's grimacing pain and tenderness over specific spinal levels. In this regard, while the patient himself refuses further diagnostic studies, his wishes about his care must be respected. CONCLUDING: However, the daughter's concerns about her father's management also need to be considered. The goal of care here is to ensure comfort and manage pain adequately, rather than curative treatment as per his wishes. RESPONSE: The most appropriate next step according to these circumstances would be to reassure the daughter and recommend hospice care consultation (Option D). End-of-life care through hospice service can effectively address the patient's physical comfort, emotional wellbeing, and ensure comprehensive pain management. It is a choice aligned with the patient's wishes while not neglecting his daughter's concerns.

**AI_E:** RESTATING THE QUESTION: This query centers around an elderly individual who previously had bilateral hip replacements and was diagnosed with prostate cancer a year ago, who is now dealing with severe lower back pain. Despite the discovery of lytic lesions and a compression fracture in his lumbosacral spine, the patient has refused further diagnostic studies. His daughter is concerned that her father will not receive adequate care due to his refusal. The question then asks, what is the next best step in alleviating the daughter's concerns. PLANNING: Clinically, the patient's refusal to undergo more diagnostic tests represents an obstacle to any attempts at therapy that might be suggested based on the results. Given his age, history of osteoarthritis, bilateral hip replacement surgery, and current symptoms alongside a refusal for further examination, several assumptions can be made. Initially, one could assume that the patient's refusal of additional investigations accounts for the daughter's distress since she may feel that her father's health state is not being fully addressed. Alternatively, the patient's prostate cancer, coupled with the radiological findings of lytic lesions and a compression fracture, could suggest metastasis causing the niece's worry, as she fears her father's health might deteriorate rapidly and uncomfortably. CLINICAL REASONING: An important clinical determinant to consider in this situation would be to address the patient's previously expressed wishes. He has made it clear that he would prefer to be kept comfortable, showing a preference for palliative care over aggressive medical treatment. As such, his symptoms, mainly severe back pain and decreased mobility likely from his metastatic disease, should be managed rather than pursuing further diagnostic tests that the patient has refused already. CONCLUDING: Given the patient’s age, pre-existing conditions, declining of further workups, and his express wishes about his care, the most appropriate step would involve a course of action directed at palliative and supportive care, rather than curative methods or in-depth diagnostic procedures. This seems to coincide with his wish to be kept comfortable. RESPONSE: Therefore, (D) "Reassure the daughter and recommend hospice care consultation" would be the most suitable next step to address the daughter's concerns while respecting the patient's previously stated preferences. This approach prioritizes the quality of the rest of the patient's life over length of life, which seems to align with his expressed wishes.

**AI_B:** RESTATING THE QUESTION: The gentleman in question is an 88-year-old male with a diagnosis of osteoarthritis and previously undergone bilateral hip replacement. However, his current complaint surrounds a 2-day history of severe low back pain that hampers his mobility, alongside a year-old diagnosis of prostate carcinoma for which he declined treatment. His current clinical examination and radiographic findings indicate a lumbosacral problem. The man's daughter is worried about her father not pursuing further evaluation and treatment. The question is asking for the most appropriate next step given the context of the patient's wishes, the daughter's concerns, and the patient's current health status. PLANNING: A preliminary assumption involves palliative care as an option. This is because of the patient's declined therapy for his prostate carcinoma and his expressed intent of just being kept comfortable. It could be that his current physical ailments are consequent of his carcinoma, which further testing might confirm. However, the patient has also expressed a refusal for any more diagnostic tests. An alternative assumption might contend with the belief that further evaluation and possible subsequent treatments could still improve the quality of this patient's life. The patient's refusal might be an obstacle but one that could be surpased with effective communication and reassurance. The selected assumption should prioritize the wishes of the patient while addressing the concerns of his daughter. CLINICAL REASONING: Given the patient's clinical history, physical examination, vital signs and radiographic findings, it's plausible that his prostate carcinoma has metastasized to his spine. This is supported by the presence of severe back pain and the discovery of lytic lesions and a compression fracture in the lumbosacral spine. Based on the patient's wish to 'die comfortably', this indicates a preference for palliative rather than aggressive treatment, which aligns with his refusal for further diagnostic studies. It's important to consider the patient's autonomy in medical decision making. CONCLUDING: Bearing in mind the patient's age, disease state, and expressed wish for comfort over aggressive treatment, recommending hospice care consultation would be the most appropriate next step. Hospice care prioritizes patient comfort, which aligns with the patient's wishes. This decision respects the patient's autonomy and takes his declining health status into consideration. In hospice care, professionals are equipped to offer pain and symptom management, as well as support services to patients and their families, providing reassurance to the patient's daughter. RESPONSE: (D) Reassure the daughter and recommend hospice care consultation.

**AI_A:** RESTATING THE QUESTION: The question presents a case of an elderly man with a history of osteoarthritis, prostate cancer, and current severe back pain that has inhibited his ability to walk. He has expressed his desire not to pursue further testing or treatment, opting instead for comfort care. The patient's daughter worries that if her father forgoes testing, he will be abandoned, and she likely desires assurance that he will continue to be cared for and that his pain will be managed. We are asked to consider what the most appropriate next step would be considering the daughter's concerns and the patient's preferences. PLANNING: There are assumptions at play in this case. The first and most apparent one is that the patient's health condition requires further testing. Another assumption could be that the daughter's concerns stem strictly from her fear for the patient's future health status rather than her understanding and acceptance of his desires for comfort care. Given that the patient has explicitly refused further diagnostic testing and has a history of prostate cancer, it is reasonable to hypothesize that the presence of lytic lesions and a compression fracture might be a result of metastatic prostate cancer. These assumptions lead us to two important considerations. Are there treatment options that could significantly improve the patient's conditions without subverting his wishes to forego extensive diagnostics? Is the daughter's primary concern about her father's comfort, or is it about neglect from doctors if her father's wishes are respected? CLINICAL REASONING: The man has decided to decline further testing and interventions, exhibiting a preference for palliative care over life-prolonging treatments. When advocating for the patient's autonomy, healthcare providers must respect his right to forgo additional diagnostic procedures, even if more aggressive treatments could be beneficial in prolonging life or improving its quality. His worsening symptoms, point tenderness over the lower back, and the imaging showing lytic lesions and a compression fracture suggest the progression of metastatic prostate cancer. Patients with terminal illnesses, such as advanced metastatic cancer, including prostate cancer, often receive benefit from hospice care, which emphasizes pain control and symptom management. CONCLUDING: We must address the daughter's concerns by ensuring her that her father will not be abandoned if he chooses not to undergo further diagnostic procedures or aggressive treatment. Hospice care offers a multidisciplinary approach that provides comprehensive management of a patient's physical, psychological, social, and spiritual needs. This service offers 24/7 availability, thus ensuring the patient and family that support and care will be provided when needed. RESPONSE: As such, in response to the daughter's concern, the most appropriate next step would be to reassure her and recommend hospice care consultation (option D). The reassurance is necessary to highlight that her father's care and comfort remains a priority despite his choice to decline further testing or aggressive treatments. The recommendation for hospice care is aligned with the patient's wishes of being kept comfortable and simultaneously addresses the daughter's fear of her father being "abandoned." Indeed, through hospice care, medical professionals can manage the patient's current symptoms and any emerging complications, emphasizing comfort and quality of life.

**Facilitator** The consensus statement synthesizes the responses of AI_A, AI_B, AI_C, AI_D, and AI_E as follows: The case presents an 88-year-old man with a history of osteoarthritis, bilateral hip replacement, and prostate cancer, who is currently experiencing severe low back pain and mobility issues. The patient has refused further diagnostic studies and expressed a desire for comfort care over aggressive treatment. His daughter, however, is concerned about her father's care and fears he will be "abandoned" if he does not pursue further evaluation and treatment. The patient's clinical history, physical examination, and radiographic findings suggest the possibility of metastatic prostate cancer, which could be causing his current symptoms. However, given the patient's explicit refusal for further diagnostic studies and his preference for palliative care, his autonomy and wishes must be respected. The daughter's concerns about her father's care are valid and need to be addressed. It is important to reassure her that her father will not be abandoned and that his comfort and quality of life remain a priority. Hospice care, which focuses on pain control, symptom management, and providing support to patients and their families, aligns with the patient's wishes and can address the daughter's concerns. Therefore, the most appropriate next step in response to the daughter's concern would be to reassure her and recommend hospice care consultation (Option D). This approach respects the patient's autonomy, addresses the daughter's concerns, and ensures the patient's comfort and quality of life.
